# Effect of Yellow Propolis on Biocompatibility of Cements: Morphological and Immunohistochemistry Analysis

**DOI:** 10.1055/s-0041-1731888

**Published:** 2021-08-24

**Authors:** Izaura Helena Chaves de Meneses, Gêisa Aiane de Morais Sampaio, Rayssa Amaral Vieira, Márcio José da Silva Campos, Polliana Muniz Alves, Matheus Melo Pithon, Rogério Lacerda-Santos

**Affiliations:** 1 Department of Dentistry, Dental School, Uniesp Centro Universitario, Cabedelo, Paraíba, Brazil; 2 Department of Dentistry, Dental School, University of Pernambuco, Arcoverde, Pernambuco, Brazil; 3 Graduate Program in Dentistry, Dental School, Federal University of Juiz de Fora, Juiz de Fora, Minas Gerais, Brazil; 4 Department of Pathology, Dental School, State University of Paraíba, Campina Grande, Paraíba, Brazil; 5 Department of Orthodontics, State University of the Southwest of Bahia, Jéquie, Bahia, Brazil; 6 Department of Orthodontics and Pediatric Dentistry, Dental School, Federal University of de Fora, Minas Gerais, Brazil

**Keywords:** microscope, propolis, biocompatibility, histological, glass ionomer cements

## Abstract

**Objective** The focus of this study was to evaluate the biocompatibility of ionomer cements modified with ethanolic extracts of propolis (EEP) in different concentrations and time intervals.

**Materials and Methods** In total, one hundred and thirty-five male Wistar rats were randomized into nine groups: Control, Groups Meron, and Groups Ketac (conventional, and added with 10, 25, 50% EEP, respectively). Histological analyses of inflammatory infiltrate and collagen fibers, and immunohistochemistry of CD68+ for macrophages (MOs) and multinucleated giant cells (MGCs) were performed.

**Statistical Analysis** Data were analyzed using the Kruskal—Wallis and Dunn ( *p* < 0.05) tests.

**Results** Intense inflammatory infiltrate was demonstrated in the cements with 10% EEP at 7 days and 15 days ( *p* < 0.05), only Group Ketac 10% EEP ( *p* = 0.01) at 30 days. A smaller quantity of collagen fibers was observed in the cements with 10% EEP ( *p* = 0.01) at 7 days, and Group Meron 10% EEP ( *p* = 0.04) at 15 days. MOs and MGCs showed significant difference for the cements with 10% EEP ( *p* = 0.01) at 7 and 15 days. At 30 days, MOs persisted in the Groups with 10% EEP.

**Conclusions** The concentration of 10% EEP had the greatest influence on the inflammatory and tissue repair processes. The concentrations of 25 and 50% EEP demonstrated biocompatibility similar to that of cements that did not receive EEP.

## Introduction

Substances such as propolis, 1 2 3 which have anti-inflammatory and antibacterial capacity, have been added to glass ionomer cements (GICs) for the purpose of improving their properties in addition to those they already have, such being capable of bonding chemically to enamel, and providing continuous release and absorption of fluoride. 4 5 6 The biological improvement of these cements may represent a significant reduction in the risk for developing caries and periodontal diseases 1 around cemented prosthetic structures and orthodontic bands. 5 7

Studies have demonstrated that modification of GICs with yellow propolis did not interfere in their mechanical properties and endowed them with antimicrobial action. 1 2 3 The use of ethanolic extract of propolis (EEP) has been an innovative strategy for incorporating antimicrobial agents with controlled release into GICs. 1 3 8 9 10 Scientific evidences have demonstrated that there is potential interest in the therapeutic use of propolis, due to its antibacterial, 1 11 12 antifungal, 13 antiviral, 14 antitumor 13 properties, and as coadjuvant action in preventing tooth enamel demineralization and gingival inflammation. 15 16

However, little is known about the biocompatibility of these modified GICs. 2 11 Studies have demonstrated that the conventional GICs are biocompatible with fibroblasts 5 and tissues, however, the modification of these cements could generate changes in the cellular inflammatory response, 2 macrophages multinucleated giant cells, 17 18 and collagenization of gingival tissue subjacent to prostheses and cemented bands. 2 In this context, it is necessary to evaluate the influence of these cements on cells, since the addition of an antibacterial agent could affect their biological properties. 15 19 Therefore, the aim of this study was to evaluate the *in vivo* biocompatibility of GICs modified with EEP in different concentrations and different time intervals.

## Material and Methods

### Ethanolic Extract of Propolis

The pure yellow propolis for use in this test was produced by bees ( *Apis mellifera ligustica* ) and was collected in João Pessoa, Brazil. Initially the propolis samples were frozen at 220°C. Afterward, the samples were ground (ZM 200, Retsch, Haan, Germany) for the purpose of obtaining a particle size of approximately 0.250 mm to increase the surface area and homogenize the sample for the process of extraction. Subsequently, the 2 g portions of samples in sterile volumetric flasks were weighed under aseptic conditions. Separately, each 2 g portion of the propolis sample was dissolved in 20 mL of 80% ethanol (vol/vol), using a mixer Shaker (MA 420, Marconi, São Paulo, Brazil) under constant agitation, at ambient temperature, for a period of 24 hours. Next, supernatant particles were removed from the EEP through a filter and the suspension was separated by centrifugation at 8,800 rpm (SIGMA 2–16 KL, Osterode am Harz, Germany) for a period of 30 minutes to produce the EEP. The samples were stored in tubes covered with aluminum foil and kept in a light-free place, at a temperature of 5°C until they were used, to prevent degradation of the material.

### Manipulation of Cements

Two GICs that contained 10% tartaric acid were used for cementation, namely: Meron-Voco (Lot-1123187, Cuxhaven, Germany) and Ketac Cem-3M/ESPE (Lot-1322600597, Seefeld, Germany) Another three solutions of yellow EEP, which contained 10, 25, or 50% of propolis in 80% alcohol, were also used to manipulate the powder of the cements tested, in a proportion of one drop of liquid (10% tartaric acid) to one drop of yellow propolis solution, using the same dosing nozzle. This portion of EEP was afterward spatulated together with the cement powder to obtain the crystallization of the material. 1

### Animal Model and Experimental Groups

For sample size calculation, a standard deviation (SD) of 2.23 and a minimal intergroup difference of 5.00 to enable the inflammatory infiltrate to be detected, five animals were required to provide a power of 80% with an *α* of 0.05. This study was approved by the Ethics Committee on Animal Research CSTR/UFCG/N.152017.

**Fig. 1:**
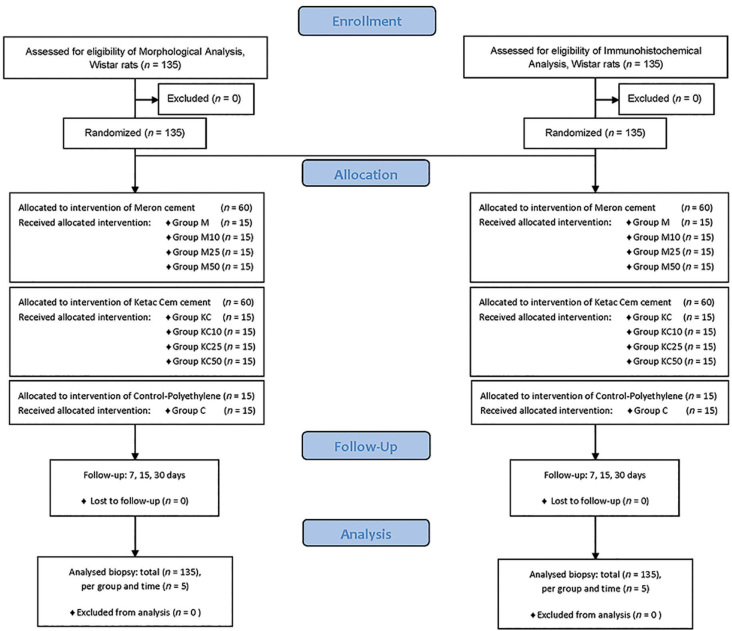
Flow diagram of animals used, groups, and tests evaluated.

In total, One hundred and thirty-five male Wistar rats (250 g) were randomized into nine groups ( *n* = 15), being: Control, Groups Meron, and Groups Ketac Cem (conventional, and added with 10, 25, 50% EEP, respectively) ( Fig. 1 ).

For the beginning of the experiments, the rats were anesthetized (sodium thiopental, 50 mg/kg, Cristália, São Paulo, Brazil). Trichotomy was performed on the dorsal region 20 21 and antisepsis with CHX 4%. 22 23

Two 8-mm long and 18-mm deep incisions were made on the back of each animal. Each rat received two polyethylene tube implants (nontoxic Scalp Vein 19G) (1.5 mm internal diameter × 5.0 mm long). The tubes were previously autoclaved at 120°C for 20 minutes and used as vehicles for the inclusion of cements.

Cements were handled following the manufacturers’ instructions and introduced into the tubes using a syringe (Centrix, Connecticut, United States) supported on a glass slide at one extremity; and a small glass slide at the other to flatten the cement surface. In the Control Group, empty polyethylene tubes were used.

After implanting the tubes in the tissue, the rats received an intramuscular injection of 0.2 mL of veterinary pentabiotic (Wyeth, New York, United States) and sodium dipyrone (0.3 mL/100 g, Novalgina, São Paulo, Brazil). Then, they were kept with appropriate food and water *ad libitum* in individual cages. 24 After the experimental periods of 7, 15, and 30 days, the rats were anesthetized to collect biopsies, then the animals were sacrificed in a CO _2_ chamber.

### Morphological Analysis

Biopsies were fixed in 10% formalin, prepared on glass slides and stained with Hematoxylin and Eosin (HE). The histological sections were evaluated in optical microscopy (DM500, Leica-Microsystems, Wetzlar, Germany), in magnifications of 100x to 400x. Inflammatory infiltrate and collagen fibers were histologically evaluated according to the scores 20 22 : 1—absent (when absent in the tissue); 2—scarce (when scarcely present, or in very small groups), 3—moderate (when densely present, or in some groups), and 4—intense (when found in the entire field, or present in large numbers). For each biopsy sample, five histological sections inserted on glass slides were analyzed. The sections were representative of the condition of the tissue adjacent to the implanted cements. 23 25 26 The microscopic evaluation in this analysis was performed by a single calibrated researcher (Kappa = 0.90).

### Immunohistochemical Analysis

The 4% formalin-fixed specimens embedded in paraffin blocks were submitted to sectioning into 3-μm thick sections that were extended on duly cleaned glass slides, defatted, and previously prepared with a 3-aminopropyltriethoxysilane-based adhesive (Sigma Aldrich Chemical, St. Louis, United States). Afterward, the material was submitted to the immunoperoxidase method by the dextran polymer technique (polymer/HRP) using anti-CD68 as primary antibody ( Table 1 ).

**Table 1:** Specificity, clone, manufacturer, dilution, antigen retrieval, and incubation time of the primary antibody used in the study

Specificity	Clone	Manufacturer	Dilution	Antigen retrieval		Incubation
CD68	ED1	Abcam	1:1,500	Citrato, pH 6, 95 degrees, 30 min	60 min	

As positive control for the CD68 antibody, subcutaneous tissue specimens from rats without insertion of the material were used, and for negative control, the primary antibody was replaced by 1% bovine serum albumin in buffer solution. The immunoreactivity was verified by the brownish coloring of the marked cells. After processing and immunohistochemical treatment of the histological sections, each specimen was analyzed under a light microscope by a previously trained and calibrated examiner (Kappa: 0.95).

In each group, 10 histological sections of the tissue adjacent to the implanted cements were analyzed. At 100x magnification, five immunoreactivity fields of the antibody were selected. At 400x magnification, each of these fields was photomicrographed (Leica DM500, Leica Microsystems, Wetzlar, DE) and the images obtained were transferred to a personal computer. With the aid of the ImageJ program (National Institute of Mental Health, United States), the CD68+ cells (MOs and multinucleated giant cells [MGCs]) were counted in each of these fields. The values obtained in each of these fields were added up, thus establishing the total number of CD68+ cells, and afterward, this datum was used to calculate the mean value for each group.

This study was randomized and triple-blind; each experimental material used in the animals was inserted in Groups I to IX, in such a way that the examiner and the statistical evaluator had no knowledge of the materials used.

## Results

### Morphological Analysis

Within 7 days, an intense inflammatory infiltrate was demonstrated, singularly in Group Meron 10% EEP and Group Ketac 10% EEP, with significant difference between the Control Group in 7 and 15 days ( *p* < 0.05) ( Table 2 ). In addition, a persistent chronic inflammatory infiltrate was observed at 30 days, with significant difference between Groups Control and Ketac 10% EEP ( *p* = 0.01) ( Table 2 ).

**Table 2:** Mean of scoresa attributed to the cements, after time interval difference

Condition time/Days	Groups									*p* **^b^**	
	M	M10	M25	M50	KC	KC10	KC25	KC50	*C*		
Inflammatory infiltrate											
7	13.75 ^AB^	20.00 ^A^	18.75 ^AB^	16.25 ^AB^	15.00 ^AB^	20.00 ^A^	18.75 ^AB^	15.00 ^AB^	10.00 ^B^		0.01
15	11.25 ^AB^	16.25 ^A^	13.75 ^AB^	11.25 ^AB^	12.50 ^AB^	16.25 ^A^	13.75 ^AB^	10.00 ^AB^	7.50 ^B^		0.01
30	10.00 ^AB^	12.50 ^AB^	12.50 ^AB^	10.00 ^AB^	10.00 ^AB^	13.75 ^A^	11.25 ^AB^	10.00 ^AB^	6.25 ^B^		0.01
Collagen											
7	12.50 ^AB^	8.75 ^A^	10.00 ^AB^	11.25 ^AB^	11.25 ^AB^	8.75 ^A^	10.00 ^AB^	11.25 ^AB^	15.00 ^B^		0.01
15	16.25 ^AB^	10.00 ^A^	16.25 ^AB^	17.50 ^AB^	16.25 ^AB^	15.00 ^AB^	17.50 ^AB^	18.75 ^B^	18.75 ^B^		0.04
30	18.75	16.25	17.50	18.75	20.00	18.75	20.00	20.00	20.00		0.14
^a^ For each sample of the study, five representative sections of the histological condition of the tissue were analyzed, when all five sections of the tissue showed the same histological condition. Scores: 1, absent (5.00); 2, scarce (10.00); 3, moderate (15.00); and 4, intense (20.00). ^b^*p* indicates nonparametric Kruskal–Wallis test, followed by Dunn’s multiple comparisons test. ^A or B^ Means followed by the same single letter did not express statistically significant difference ( *p* >0.05). ^AB^ Means followed by different letters expressed statistically significant difference ( *p* < 0.05).											

In the tissue repair events, a smaller quantity of collagen fibers was observed in Groups with 10% EEP compared with the Group Control ( *p* = 0.01) at 7 days, and smaller in Group Meron 10% EEP when compared with the Group Control and Ketac 50% EEP ( *p* = 0.04) at 15 days ( Table 2 ). In the 30-day period, the healing process was similar between the Propolis Groups and the Control Group ( *p* = 0.14).

### Statistical Analysis

For data analysis, the Kolmogorov–Smirnov test (GraphPad-Prism 5.0, San Diego, United States) was used. The histological data did not present a normal distribution and, so, the Kruskal–Wallis and Dunn nonparametric tests were used ( *p* < 0.05).

### Immunohistochemical Analysis

**Fig. 2:**
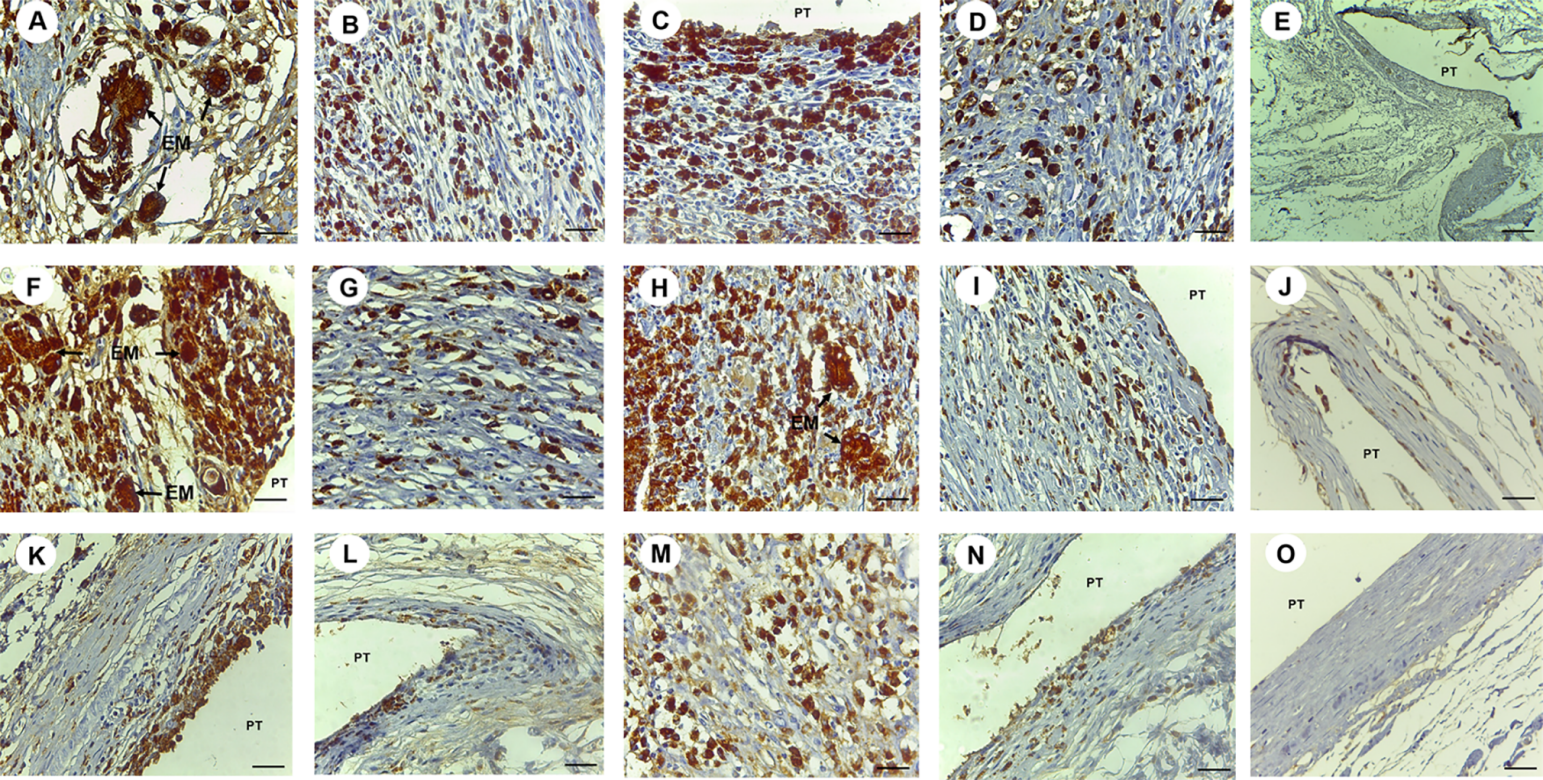
Immunomarking for antibody CD68. In time interval of 7 days: ( **A** ) In Group M10, intense immunomarking of CD68 was noted, with brownish cytoplasm immunopositive for multinucleated giant cells (MGCs) and macrophages (MOs) close to the exogenous material (EM) enveloped by MGCs (IH, 400X magnification; scale: 25 µm). ( **B** ) In Group M50, moderate immunomarking of CD68 was observed for MGCs and MOs (IH, 400X magnification; scale: 25 µm). ( **C** ) In Group KC10, intense immunomarking of CD68 was observed, with brownish cytoplasm immunopositive for MGCs and MOs throughout the cavity with polyethylene tube implant (PT) (IH, 400X magnification; scale: 25 µm). ( **D** ) In Group KC50, moderate immunomarking of CD68 was observed for MGCs and MOs (IH, 400X magnification; scale: 25 µm). ( **E** ) In Group Control, absence of immunoreactivity to CD68 was observed for MGCs and MOs around the cavity with PT (IH, 100X magnification; scale: 100 µm). *In time interval of 15 days:* ( **F** ) In Group M10, intense immunomarking of CD68 was noted, with brownish cytoplasm immunopositive for MGCs and MOs with immunopositive brownish cytoplasm close to the EM and around the cavity with PT (IH, 400X magnification; scale: 25 µm). ( **G** ) In Group M50, low immunomarking of CD68 was observed for MGCs and MOs (IH, 400X magnification; scale: 25 µm). ( **H** ) In Group KC10, intense immunomarking of CD68 was observed for MGCs and MOs with immunopositive brownish cytoplasm close to EM (IH, 400X magnification; scale: 25 µm). ( **I** ) In Group KC50, low immunomarking of CD68 was observed for MGCs and MOs surrounding cavity with PT (IH, 400X magnification; scale: 25 µm). ( **J** ) In Group Control, absence of immunoreactivity to CD68 was observed for MGCs and MOs surrounding cavity with PT (IH, 200X magnification; scale: 50 µm). *In time interval of 30 days* : ( **K** ) In Group M10, moderate immunomarking of CD68 was noted, with brownish cytoplasm immunopositive for MGCs and MOs in the internal region of the cavity with PT (IH, 400X magnification; scale: 25 µm). ( **L** ) In Group M50, scarce immunomarking of CD68 was observed for MGCs and MOs surrounding cavity with PT (200X magnification; scale: 50 µm). ( **M** ) In Group KC10, moderate immunomarking of CD68 was observed, with brownish cytoplasm immunopositive for MOs and low quantity of MGCs surrounding the cavity (IH, 400X magnification; scale: 25 µm). ( **N** ) In Group KC50, scarce immunomarking of CD68 was observed for MGCs and MOs surrounding cavity with PT (IH, 200X magnification; scale: 50 µm). ( **O** ) In Group Control, absence of immunoreactivity to CD68 was observed for MGCs and MOs surrounding the cavity with PT (IH, 400X magnification; scale: 25 µm).

In the immunohistochemical analysis, the MGCs demonstrated significant difference between the Control Group when compared with Groups M10 and KC10 ( *p* = 0.01) in the time intervals of 7 ( Fig. 2A–E ) and 15 days ( Fig. 2F–J ). In the time interval of 30 days only Group M10 showed statistically different quantities of cells when compared with the Control Group ( *p* = 0.01) ( Fig. 2K–O ; Table 3 ).

**Table 3:** Immunohistochemical analysis of the quantity of multinucleated giant cells and macrophages, after the time intervals of 7, 15, and 30 d

Condition time/Days	Groups										
	M	M10	M25	M50	KC	KC10	KC25	KC50	C		*p* **^b^**
Multinucleated giant cells											
7	1.00 ^AB^	5.00 ^A^	2.00 ^AB^	1.00 ^AB^	1.00 ^AB^	4.00 ^A^	2.00 ^AB^	1.00 ^AB^	0.00 ^B^		0.01
15	0.00 ^A^	3.00 ^B^	1.00 ^AB^	1.00 ^AB^	0.00 ^A^	3.00 ^B^	1.00 ^AB^	0.00 ^A^	0.00 ^A^		0.01
30	0.00 ^A^	2.00 ^B^	0.00 ^A^	0.00 ^A^	0.00 ^A^	1.00 ^AB^	1.00 ^AB^	0.00 ^A^	0.00 ^A^		0.01
Macrophages											
7	46.40 ^A^	91.62 ^B^	71.90 ^AB^	54.67 ^AB^	61.05 ^AB^	83.35 ^B^	67.90 ^AB^	54.35 ^AB^	22.21 ^A^		0.01
15	55.35 ^AB^	98.57 ^A^	77.92 ^AB^	50.42 ^B^	69.10 ^AB^	97.95 ^A^	74.72 ^AB^	59.70 ^AB^	14.82 ^B^		0.01
30	42.17 ^A^	67.92 ^B^	61.05 ^AB^	47.07 ^AB^	52.30 ^AB^	72.20 ^B^	63.00 ^AB^	57.12 ^AB^	11.45 ^A^		0.01
Note: These values represent the mean quantity of cells found in the histological sections representative of the tissue evaluated ( *n* = 10 per group). ^b^*p* indicates nonparametric Kruskal–Wallis test, followed by the Dunn multiple comparisons test. ^A or B^ Means followed by the same single letter did not express statistically significant difference ( *p* >0.05). ^AB^ Means followed by different letters expressed statistically significant difference ( *p* <0.05).											

A higher number of MOs were demonstrated in the Groups with 10% propolis. There was a significant difference between the Control Group when compared with Groups M10 and KC10 ( *p* = 0.01) in the time intervals of 7 ( Fig. 2A–E ) and 15 days ( Fig. 2F–J ). A reduction in the quantity of MOs was demonstrated over the course of the experimental time intervals, however, this type of cell still persisted significantly in Groups M10 and KC10 in the time interval of 30 days ( *p* = 0.01) ( Fig. 2K–O ). The quantity of MOs was less significant in Groups M25, M50, KC25, and KC50 and was shown to be similar to the quantity in the Control Group ( Table 3 ).

## Discussion

Propolis has been widely used in the field of health care, due to its antibacterial, 2 12 and anti-inflammatory capacity, 27 among other characteristics. 13 14 28 Furthermore, studies have demonstrated that the addition of propolis did not modify the physicomechanical properties of the GICs, 1 2 3 nevertheless little is known about its influence on tissues.

EEP has aromatic fatty acids and phenolic compounds in its molecules. These polyphenols 29 have structures that favor improvement in the mechanical properties of GICs, 2 by means of the chelation reaction between the phenolic groups of hydroxyl and carboxyl of GIC, 30 thereby providing a larger quantity of polysalts for binding sites. 31

The first biocompatibility analysis was performed after 7 days, as it is only after this period that a more organized inflammatory reaction can be expected. 32 Intense inflammatory infiltrate was demonstrated only in the cements with 10% EEP in the time intervals of 7 and 15 days ( *p* = 0.01). The intensity of the inflammatory infiltrate was shown to be inversely proportional to the experimental time intervals and concentration of propolis; however, a persistent, chronic infiltrate was exhibited in the Ketac cement 10% EEP ( *p* = 0.01) at 30 days. Although the inflammatory reaction may be influenced by the release of small quantities of aluminum and/or iron ions present in the composition of the GICs, capable of causing oxidative stress in the cells and interfering in the cellular response, 26 this influence did not appear to be significant with the addition and synergism of the EEP in GIC.

The low concentration of EEP in the cements with 10% allied to the presence of alcohol that functioned as solvent or vehicle for the propolis, 33 may have generated a low potential for rapid tissue healing. 2 At higher concentrations of EEP such as 50%, improved anti-inflammatory and healing effects were demonstrated, 1 which suggested that higher concentrations of EEP would be capable of diminishing the potentially aggressive effect of the alcohol on the tissues, 2 34 thereby potentiating the anti-inflammatory effect of propolis by means of the mechanism of action of the flavanone pinocembrin, flavonoid galangin, and caffeic acid phenethyl ester 35 36 ; the antibacterial effect through inhibition of bacterial RNA polymerase 3 is particularly significant in the concentrations of 25 and 50%. 1 3 Moreover, authors have demonstrated an antiadherent activity of GIC with EEP, which may be linked to the changes in the hydrophobic bond of this association. 37 This may also have contributed to the low inflammatory response demonstrated in this study.

The purpose of the analysis after 30 days was to verify the repair by collagenization after the initial aggression. 10 A smaller quantity of collagen fibers was observed in the cements with 10% EEP ( *p* = 0.01) at 7 days, and this was lower in the Meron cement 10% EEP ( *p* = 0.04) at 15 days, without difference in healing compared with Control at 30 days ( *p* = 0.14). Researches have demonstrated that propolis in different final physical stages may interfere in cell viability 36 38 39 and reduce the production of noncollagen protein and collagen fibers. 26 In this study, the tissue behavior of GIC with EEP appeared to allow a slow and continuous release of propolis into the medium, due to the solid, porous, polymeric chain of the conventional ionomers. 40 This is in agreement with the findings of this study, in which the cements with higher concentrations of EEP showed larger numbers of collagen fibers and healing throughout the experiment; and a concentration-dependent relationship of EEP with the tissue healing process. 2 10

The use of immunohistochemistry with CD68 for MOs and MGCs, was capable of offering more precise results relative to the inflammatory response. 17 41 A larger number of MOs were demonstrated in the Groups with 10% EEP in the time intervals of 7 and 15 days ( *p* = 0.01). A reduction in the quantity of MOs was demonstrated over the course of the experimental time intervals, however, there was still significant persistence of the quantity of MOs in the cements with 10% EEP in the time interval of 30 days ( *p* = 0.01). There was less significant presence of MOs in the cements with 25 and 50% EEP, without differing from the Control. MOs are responsible for potentiating the proinflammatory response 41 ; they act in the presentation of antigens, phagocytosis, recruitment of fibroblasts, 42 degradation and/or isolation of the cement 10 and substances of a toxic nature. 43 The effects of the factor cement, its components, and alcohol on tissue response, appear to have been more significant in the cements with 10% EEP, because at the higher concentrations of EEP the inflammatory and healing conditions were similar to those of the Controls.

As the particles released by the GICS have difficulty in being digested by the MOs, they fuse and form a larger number of phagocytic cells from the MGCs, for the purpose of facilitating degradation 22 26 44 of the cement rests. The increase in quantity of MGCs in the tissues reflected the efforts of the organism to isolate and degrade the material more efficaciously 22 and rapidly.

A larger quantity of MGCs were demonstrated in the cements with 10% EEP ( *p* = 0.01) in the time intervals of 7 and 15 days. A gradual reduction in the quantity of MGCs occurred in the experimental time intervals, although the cement M10 still presented quantities that differed from those of the Control ( *p* = 0.01) at 30 days. The presence and persistence of GMCs 10 18 45 in the cements with 10% EEP may have been linked to the higher number of MOs recruited and the organism’s need to isolate the foreign body. 10 Considered in conjunction, propolis was shown to have satisfactory tissue biocompatibility, in which its anti-inflammatory and healing effects in the higher concentrations were capable of exceeding its foreign body effect, presence of metal ions, and alcohol. Its biological effect has the potential to inflammatory control and repair of gingival tissues in different clinical conditions. 46 47 Clinical experiments with humans could check the efficacy of what appears to be a highly promising method for obtaining an antibacterial GIC.

## Conclusions

- The histocompatibility analysis showed that the intensity of histological changes in the cements were inversely proportional to the concentration of propolis added.
- The concentration of 10% EEP had the greatest influence on the inflammatory and tissue repair processes.
- The concentrations of 25 and 50% EEP demonstrated biocompatibility similar to that of cements that did not receive EEP.
